# Plasma Ammonia Levels in Newborns with Asphyxia

**Published:** 2016

**Authors:** Nasrin KHALESSI, Nastaran KHOSRAVI, Maryam MIRJAFARI, Ladan AFSHARKHAS

**Affiliations:** 1 Department of Pediatric Neonatology, Ali-Asghar Children’s Hospital, Iran University of Medical Science, Tehran, Iran; 2 Pediatrician, Ali-Asghar Children’s Hospital, Iran University of Medical Science, Tehran, Iran; 3 Department of Pediatric Neurology, Ali-Asghar Children’s Hospital, Iran University of Medical Science, Tehran, Iran

**Keywords:** Asphyxia, Hyperammonemia, Neonates

## Abstract

**Objective:**

Perinatal asphyxia may result in hypoxic damage in various body organs, especially in the central nervous system. It could induce cascade of biochemical events leading to the cell death and metabolic changes, eventually may increase plasma ammonia levels. The purpose of this study was to determine the prevalence of hyperammonemia in neonates with asphyxia and to find the relationship between ammonia levels and severity of asphyxia.

**Material & Methods:**

In this cross-sectional study, we included 100 neonates with perinatal asphyxia in the Neonatal Intensive Care Unit of Ali-Asghar Hospital, Iran University of Medical Science, Tehran, Iran in 2010-2011. All full term patients diagnosed of asphyxia were enrolled. The relationship between plasma ammonia levels and sex, gestational age, birth weight and severity of asphyxia were determined. Data were analyzed using SPSS software.

**Results:**

Fifty six percent of neonates were male. The mean gestational age was 38.0± 1.2 wk. Mean plasma ammonia level was 222 ± 100 μg/dl and 20% of the neonates had hyperammonemia. It was not associated with gender, gestational age, birth weight, and asphyxia severity. Six patients died and mean plasma ammonia levels was 206±122 μg/dl. In this group, there was no significant relation between plasma ammonia levels and severity of asphyxia. No significant different was seen between plasma ammonia in dead and lived neonates.

**Conclusion:**

According to high prevalence of hyperammonemia in neonatal asphyxia, measurement of plasma ammonia levels, is suggested to improve management of asphyxia.

## Introduction

In human body, amino acid metabolism produces ammonia. It is mostly in form of ammonium at physiologic pH and maintained at low concentration by urea cycle function that converts ammonium to urea in the liver and excretes it in urine (1). In the central nervous system (CNS), urea cycle is not active and glutamine synthetase of asterocytes converts the glutamate and ammonium to glutamine (2). In newborns, hyperammonemia is potentially fatal for developing brain by increasing lactate, compromised energy metabolism, brain edema and disturbances in electrophysiology of the neurons (3). It may lead to loss of consciousness, seizures, abnormal muscle tone and cognitive impairment. In pediatric age groups, hyperammonemia can cause cortical atrophy, demyelination and ventricular enlargement. Extension of damages depends on duration and severity of the exposure to ammonium (1, 4, 5). Hyperammonemia in neonates has various inherited and acquired etiologies. Inborn errors of metabolism, including urea cycle disorders, organic acidemias and fatty acid oxidation disorders are inherited causes of hyperammonemia (5, 6). Transient hyperammonemia of the newborn (THAN) is a condition with severe hyperammonemia. The possible pathogenesis of THAN is transient platelet activation and shunting of blood from the portal circulation of the liver to the systemic circulation (7, 8). Other conditions with hyperammonemia are liver dysfunction associated with sepsis, herpes simplex infection and perinatal asphyxia (8, 9). Asphyxia usually occurs in term infants (gestational age more than 37 wk) who have signs of fetal distress before delivery and low apgar scores and may develop neurologic complications in 24 h after delivery (10). Perinatal asphyxia can cause hypoxic damage in various organs, including kidneys, lungs, liver, and most importantly CNS. At the cellular level, hypoxic– ischemic damage may create a cascade of biochemical events leading to the cell death (3). During hypoxic stress, protein breakdown may increase and hepatic urea biosynthesis decrease and lead to hyperammonemia (11). Rising of the plasma ammonia in neonates suffered from asphyxia, could be associated with further increase the risk of brain damage (1). Accordingly, assessment of hyperammonemia in cases of perinatal asphyxia seems to have a great importance in management of the patients. The present study was conducted to evaluate the prevalence and associated factors of hyperammonemia in newborns with perinatal asphyxia.

## Materials & Methods

This prospective cross sectional study was performed in Neonatal Intensive Care Unit (NICU) of Ali-Asghar Tertiary Hospital, Iran University of Medical Science, Tehran, Iran, from January 2010 to April 2011. The study was approved by Medical Ethics Committee of Iran University of Medical Sciences. Informed consent was taken from parents and the cases enrolled in the survey. During the study period, 102 patients of 1-6 h age, with diagnosis of asphyxia were enrolled. Definitive diagnosis of perinatal asphyxia was according to the following criteria: symptoms of encephalopathy (hypotonia, reduced reflexes, changes in pupils, and seizure), apgar score of 0 to 3 for longer than 5 min, umbilical cord arterial pH less than 7, and multisystemic organ dysfunction (12). In our cases, asphyxia was determined by a neonatologist who referred neonates from Akbarabadi Maternity Hospital to our center. In Ali-Asghar NICU, patients were visited by second neonatologist and re-evaluated for severity of asphyxia according to Sarnat and Sarnat classification. Perinatal asphyxia from point of severity has three stages: In stage 1 there are mild encephalopathy, infant hyperalertness, dilated pupils and normal electroencephalography (EEG). Stage 2 includes moderate encephalopathy, lethargy, hypotonia, small pupils, seizures and abnormal EEG. In stage 3, there are severe encephalopathy, stupor, flaccidity, absence of reflexes, seizures and abnormal EEG with decreased background activity (13). Exclusion criteria were defined as preeclampsia, neonatal systemic infections, prematurity, infants of diabetic mothers and metabolic disorders. Two subjects with underlying metabolic diseases-urea cycle enzyme defects, which developed hyperammonemia, also were excluded. During the first 24 hours of the birth before initiating hyperalimentation, venous blood samples were taken and 2 ml blood collected on EDTA and transferred with dry ice. Then plasma ammonia levels were measured by colormetric method (Cobas Integra Auto analyzer, Germany). Values of 50- 100 μg/dl as normal ranges of plasma ammonia (for full term neonate) and values above 150 μg/dl as hyperammonemia were defined (14). After detection of hyperammonemia in each case, treatment was started immediately using sodium benzoate, 250 mg/kg as bolus dose during 90-120 min and 250 mg/kg for 24 h intravenous infusion as routine protocol of treatment for hyperammonemia in our center. Plasma ammonia were checked daily and after reaching to normal levels, benzoate infusion was tapered. Daily follow up and management of patients with hyperammonemia was performed by the neonatologist. In all patients with hyperammonemia, treatment with sodium benzoate was continued for 3-7 d until normalizing of plasma ammonia levels. Data about gestational age, divided to four groups of 37, 38, 39 and 40 wk, sex, birth weight, and asphyxia severity were analyzed using SPSS 13 software (Chicago, IL, USA). Student’s t-test, ANNOVA, Chi-2, and Pearson’s correlation were performed for analysis.

**Table 1:** Clinical Characteristics of All Neonates with Perinatal Asphyxia

**Variables **	**Numbers**
**Gestational Age (weeks) **	38.0±1.2
**Gender**	
Male Female	56% 44%
**Birth weight (gr**)	2753± 512
**Ammonia level (mg/dl) **	222 ± 100
**Asphyxia severity**	
Stage 2 Stage 3	58% 42%

**Table 2:** Characteristics of Asphyxiated Newborns with and without Hyperammonemia

**Variable **		**Hyperammonemia** **[n=20] n (%)**	**Normal ammonia** **[n=80] n (%)**	***P*** **-value**
**Gestational Age (weeks)**	37 38 39 40	11 (55) 3 (15) 1(5) 5 (25)	39 (49) 14 (17) 12 (15) 15 (19)	0.63
**Gender**	Male Female	10 (50) 10 (50)	46 (57.5) 34 (42.5)	0.54
**Asphyxia severity**				
** Stage** ** Stage**	2 3	12 (60) 8 (40)	46 (57.5) 34 (42.5)	0.35
**Death**				
** Stage 2 ** ** Stage 3**		1(5) 3(15)	1(1.25) 1(1.25)	0.5

## Results

Among 100 neonates with perinatal asphyxia, 56% were male. Mean gestational age was 38.0 ± 1.2 wk (rage 37 to 40 wk). Birth weights were between 1670 gr and 4080 gr (mean 2753 ± 512 gr). Fifty-eight patients had stage2 of asphyxia and 42 cases stage 3. Mean plasma ammonia level was 222 ± 100 μg/dl. Twenty percent of cases had hyperammonemia (Plasma ammonia levels more than 150 μg/dl). After consultation with an endocrinologist, it was treated. There was no significant difference between boys and girls in terms of plasma ammonia levels (217 ± 100 μg/ dl vs. 229 ± 100 μg/dl, respectively; P=0.58). Besides, no significant difference was seen between plasma ammonia levels and gestational age (P=0.50). Mean plasma ammonia level was 199 ± 70 μg/dl in neonates with stage 2 of asphyxia, and 209 ± 70 μg/dl in stage 3 asphyxia cases. However, this difference was not statistically significant (P=0.54). The patients were divided into groups with and without hyperammonemia and were compared. Hyperammonemia was not related to sex, birth weight, and asphyxia severity in two groups (Table 2). Six patients died and mean plasma ammonia levels was 206±122 μg/dl. In this group, there was no significant relation between plasma ammonia level and severity of asphyxia (P value = 0.5) and no difference between plasma ammonia in dead and lived neonates as well (P value=0.18).

## Discussion

Hyperammonemia is a medical emergency that may lead to coma. Rapid management is essential to achieve the desire results. Although severe hyperammonemia for more than 24 h is associated with an irreversible damage of nervous system, probably the duration of hyperammonemia is more important than the level of plasma ammonia. Neonatal hyperammonemia may develop due to inborn errors of metabolism - such as urea cycle enzyme defects and organic acidemias, hepatic failure, portal systemic encephalopathy, hypovolemic shock, prematurity, respiratory distress syndrome and asphyxia (11, 15). Elevating plasma ammonia levels during hypoxic stress are ascribed to increased protein breakdown and decreased ammonia clearance in the liver (16). Hyperammonemia occurs more in severely asphyxiated newborn and lead to CNS disruption that can be associated with neurologic complications such as seizure and coma (11, 14). In our study, plasma ammonia levels were evaluated in neonates with perinatal asphyxia, and hyperammonemia was detected in 20% of subjects. There was no association between gender, gestational age, birth weight, asphyxia severity and plasma ammonia levels. From 82 high-risk neonates, 19 (23%) had hyperammonemia, which was higher in asphyxiated infants and there was no correlation between plasma ammonia levels and birth weight (14). Goldberg et al. studied 12 infants with severe perinatal asphyxia who had hyperammonemia (302-960 μg/ dl). The survivors had severe neurologic dysfunction that suggested clinical entity secondary to perinatal hyperammonemia (11). Beddis et al. measured plasma ammonia in 42 neonates admitted NICU with perinatal asphyxia and hyaline membrane disease. One hundred and two specimens were taken within the first three weeks of life. Twenty-seven cases had birth asphyxia. Mean plasma ammonia levels was 132.3 μg/dl with ranges from 44.8 to 357 μg/dl (17). Although plasma ammonia levels in their study was above normal ranges especially in asphyxiated cases, It was lower than our findings that may be due to their small size study, heterogenesity of cases and their different technique of ammonia measurement and equipment. We treated all patients with hyperammonemia. As short outcome, six patients died because of severe hypoxic brain damage and not due to hyperammonemia. Although 4 cases of dead neonates had hyper- ammonemia, there was no significant difference between plasma ammonia levels in dead and lived neonates. It means that treatment of hyperammonemia may improve prognosis, but other causes such as severe hypoxic brain damage, lead to death in asphyxiated neonates, which are unavoidable. According to our findings, hyperammonemia and asphyxia may be concomitant and it can cause further damage of the nervous system in the patients. It is not routine to do metabolic screening for neonates after birth in our country. Therefore, it is recommended to evaluate plasma levels of ammonia in asphyxiated neonates, for early diagnosis and appropriate management of hyperammonemia and it may improve some neurologic manifestations in these patients. However, there are some cases of inborn error of metabolism such as two cases with urea cycle enzyme defects with asphyxia and hyperammonemia admitted but excluded from our study. These cases must be evaluated with metabolic screening before diagnosis of transient hyperammonemia. We had some limitations including not having obstetric ward in our center and not able to preciously evaluate the patients and observe them during the labor. Besides, we could not have two hyperammonemic groups with and without treatment for comparison.

## Notes

**Authors’ contribution** Dr Khalessi has designed the research and Dr Mirjafari collected data. Dr Khalessi, Dr Khosravi, Dr Mirjafari and Dr Afsharkhas have contributed in writing the article. Dr Afsharkhas has also analyzed data and edited the paper.

## References

[1] Brar G, Thomas R, Bawle EV, Delaney-Black V (2004). Transient hyperammonemia in preterm infants with hypoxia. Pediatr Res.

[2] Haberle J (2013). Clinical and biochemical aspects of primary and secondary hyperammonemic disorders. Arch Biochem Biophysics.

[3] Konecki UL, Batshaw ML, Swaiman KF, Ashwal S, Ferrio DM, Schor NF (2012). Inborn Errors of Urea Synthesis. Swaiman’s Pediatric Neurology principle and practice.

[4] Joseph M, Hageman J R (2002). Neonatal Transport: A 3-Day- Old Neonate with Hypothermia, Respiratory Distress, Lethargy and Poor Feeding. J Perinatol.

[5] Enns G M (2005). Inborn Errors of Metabolism Masquerading as Hypoxic-Ischemic Encephalopathy. Neo Rev.

[6] Rezvani I, vFicicioglu CH, Kliegman RM, Stanton BF, St Geme JW, Schor N (2015). Defects in Metabolism of Amino Acids. Nelson textbook of pediatrics.

[7] Jain S, Chawla D (2010). Transient hyperammonemia of newborn. Dicle Med J Cilt.

[8] Chung MY, Chen CC, Huang LT, Ko TY, Lin YJ (2005). Transient hyperammonemia in a neonate. Acta Pediatr Taiwan.

[9] Burton B (1998). Inborn Errors of Metabolism in Infancy: A Guide to Diagnosis. Pediatrics.

[10] Golubnitschaja O, Yeghiazaryan K, Cebioglu M, Morelli M, Marschitz M H (2011). Birth asphyxia as the major complication in newborns: moving towards improved individual outcomes by prediction, targeted prevention and tailored medical care. EPMA J.

[11] Goldberg RN, Cabal LA, Sinatra FR, Plajstek CE, Hodgman JE (1979). Hyperammonemia associated with perinatal asphyxia. Pediatrics.

[12] Leuthner S R, Das U G (51). Low Apgar scores and the definition of birth asphyxia. Pediatr Clin N Am.

[13] Sarnat HB, Sarnat MS (1976). Neonatal encephalopathy following fetal distress. A clinical and electroencephalographic study. Arch Neurol.

[14] Sakaguchi Y, Yuge K, Yoshino M, Yamashita F, Hashimoto T (1982). Hyperammonemia in the neonate with hypoxia. Adv Exp Med Biol.

[15] Ballard RA, Vinocur B, Reynolds J W, Wennberg RP, Merritt A, Sweetman L, Nyhan W L (1978). Transient hyperammonemia of the preterm infant. N Engl Med J.

[16] Hwang MW, Yu ST, Oh YK (2010). A case of severe transient hyperammonemia in a newborn. Korean J Pediatrics.

[17] Beddis IR, Hughes EA, Rosser E, Fenton JC (1980). Plasma ammonia levels in newborn infants admitted to an intensive care baby unit. Arch Dis Child.

